# A novel mast cell marker gene-related prognostic signature to predict prognosis and reveal the immune landscape in head and neck squamous cell carcinoma

**DOI:** 10.3389/fimmu.2025.1538641

**Published:** 2025-07-09

**Authors:** Yingmiao Lin, Fangcai Wu, Xuchun Huang, Zhihan Zhang, Cantong Liu, Yiwei Lin, Yiwei Xu, Haipeng Guo, Chaoqun Hong

**Affiliations:** ^1^ Department of Clinical Laboratory Medicine, Cancer Hospital of Shantou University Medical College, Shantou, China; ^2^ Department of Radiation Oncology, Cancer Hospital of Shantou University Medical College, Shantou, China; ^3^ Department of Head and Neck Surgery, Cancer Hospital of Shantou University Medical College, Shantou, China; ^4^ Department of Oncological Laboratory Research, Cancer Hospital of Shantou University Medical College, Shantou, China

**Keywords:** mast cell, head and neck squamous cell carcinoma, single-cell sequencing, immune infiltration, immunotherapy

## Abstract

**Background:**

Head and neck squamous cell carcinoma (HNSCC) is a highly aggressive and heterogeneous malignant tumor. Mast cells are one of the immune cells widely distributed in the tumor microenvironment (TME), and their immune response with various immune cells is essential in promoting or inhibiting tumor growth and metastasis. However, the role played by mast cells in HNSCC has yet to be fully clarified.

**Methods:**

We identified mast cell marker genes using single-cell RNA sequencing (scRNA-seq) from the GSE103322 of the GEO database. The HNSCC data from the TCGA databases was divided into training and validation groups. Cox regression and LASSO regression analyses were used to screen the prognostically relevant mast cell-related genes (MRGs) to construct a prognostic signature and differentiate risk groups. The receiver operating characteristic (ROC) and calibration curves were used to test the model’s accuracy. We revealed the immune landscape of HNSCC by immune infiltration, immune checkpoint levels, ESTIMATE, and TIDE analyses. Drug sensitivity analyses were used to understand the sensitivity of different risk groups to drug therapy.

**Result:**

The 14-MRGs prognostic signature classified patients into high- and low-risk groups, and the overall survival (OS) of the low-risk group was significantly higher than that of the high-risk group (p < 0.05). The areas under the ROC curves of the nomogram were 0.740, 0.737 and 0.707 at 1-, 3-, and 5-year, and they also showed better detection efficacy in the validation group than other independent predictors. The low-risk group had richer immune cell infiltration and higher immune scores. The lower TIDE score in the low-risk group demonstrates that patients in this group were less prone to have immune escape and more likely to benefit from immunotherapy. In addition, the low-risk group was more sensitive to a broader range of drugs than the high-risk group.

**Conclusion:**

We combined scRNA-seq data and bulk RNA-seq data to construct a 14-MRGs-based prognostic model capable of well predicting the prognosis of HNSCC patients. This model may also help identify patients who can benefit from immunotherapy.

## 1 Introduction

Head and neck squamous cell carcinoma (HNSCC) is a malignant tumor that occurs in the oral mucosal epithelium, nasopharynx, oropharynx, hypopharynx and larynx and accounts for 90% of head and neck tumors (1). Globally, there are approximately 890,000 new cases and 450,000 deaths of HNSCC in 2022, and the incidence is expected to increase by 30% by 2030 (1, 2). The highly aggressive nature of HNSCC makes it a considerable challenge to treat. In the past two decades, immune checkpoint inhibitors (ICIs) have opened new horizons and become one of the main cancer treatment methods (3). In particular, the discovery of CTLA-4 and PD-1/PD-L1, as well as the molecular targeted therapies pembrolizumab and navulizumab, have improved the overall survival of patients with metastatic or recurrent HNSCC (4, 5). However, due to tumor heterogeneity and differences in patient response to ICIs therapy, a minority of patients benefit in the long term. The objective remission rates (ORR) of advanced patients are only 15-23% (4–6), and about 60% develop resistance to immunotherapy (7). Therefore, identifying reliable biomarkers to predict patient survival and find a suitable treatment for patients is essential to improve survival and outcomes.

In recent years, mast cells have received increasing attention due to their dual role in promoting tumor development and enhancing anti-tumor immunity, which may be closely related to tumor type, mast cell distribution, tumor stage, mast cell status, and their interactions with other immune cells in TME (8). Studies have shown that mast cells exhibit different functions in various cancers, such as lung, breast, renal, and prostate (9–13). In HNSCC, the role of mast cells varies depending on the specific conditions of the TME, and existing research results show significant contradictions (14). Liang et al.’s study found that mast cell infiltration was more abundant in HNSCC tumor tissues than adjacent non-tumor tissues (15). However, another study indicated that mast cells in OSCC tumor tissues were reduced compared to normal tissues (16). Additionally, the distribution of mast cells in HNSCC may be related to specific tissue sites and influenced by external factors. Cosoroabă et al.’s research found that mast cell enrichment was more significant in squamous cell carcinoma (SCC) of the lip than the tongue, pharynx, and larynx, possibly due to long-term sunlight exposure inducing mast cell recruitment (17). Mast cell infiltration may also be related to the tumor stage of HNSCC and affect patient survival prognosis. Jin et al. observed that resting mast cell infiltration significantly decreased in advanced T-stage HNSCC and speculated that resting mast cells might suppress HNSCC progression (18). Brockmeyer et al. showed that patients with high mast cell density in tumor-associated stroma had longer OS. Mast cells in different states, such as resting or active, also potentially impact the progression of HNSCC and the survival prognosis of patients. Jin et al.’s study further indicated that a high abundance of activated mast cells was associated with poorer OS in the high-risk group (19). In studies constructing prognostic models for OS in HNSCC patients, Ding et al. and Fan et al. also reached similar conclusions (20, 21), suggesting that activated mast cells may potentially promote the progression of HNSCC. However, some scholars hold different views. Chen et al. found in their study constructing a risk model of senescence-associated genes in HNSCC that the high-risk group had a higher proportion of resting mast cells and lower levels of infiltrating activated mast cells (22), and Tao et al.’s study also confirmed this finding (23). Thus, the impact of mast cells in different states on the occurrence, development, and survival prognosis of HNSCC needs to be comprehensively analyzed in conjunction with the specific conditions and multiple factors in the TME.

Mast cells participate in and regulate the biological activities of other immune cells by releasing bioactive mediators, thereby affecting the progression of tumors. For example, histamine promotes the shift in the Th1 and Th2 ratio by increasing the intracellular cyclic adenosine monophosphate content, suppressing the Th1 phenotype and enhancing the Th2 phenotype, thereby promoting tumor development (24). Furthermore, the synapse-like structures formed between mast cells and dendritic cells facilitate antigen transfer and promote T cell activation, enhancing anti-tumor immunity (25). In summary, the role of mast cells in the occurrence and development of HNSCC is diverse and complex, and further exploration is needed.

The appearance of single-cell RNA sequencing (scRNA-seq) technology could assist us in characterizing tumor cells, immune cells, and stromal cells at the level of cellular resolution, describing the tumor heterogeneity (26), which has helped us to study cell clusters and their subgroups. In this study, we integrated scRNA-seq and bulk RNA-seq data from public datasets to construct a prognostic model based on mast cell-related genes (MRGs), predict patient outcomes, and reveal the immune landscape of HNSCC to identify patients who may benefit from immunotherapy.

## 2 Materials and methods

### 2.1 Data source

The scRNA-seq data containing 5902 cells from 21 HNSCC patients were downloaded from the GSE103322 of the Gene Expression Omnibus (GEO, https://www.ncbi.nlm.nih.gov/geo/) database. Bulk-RNA-seq data and clinical information were obtained from the Cancer Genome Atlas (TCGA, https://portal.gdc.cancer.gov/) database, and data of 516 HNSCC patients with complete survival status and clinicopathological information were retained. To minimize the influence of irrelevant factors arising from the original count values, we converted the count data from TCGA to TPM data and performed a log2 (TPM+1) transformation. Additionally, we used the “removeBatchEffect” method to eliminate batch effects among samples from different sites.

### 2.2 Quality control of scRNA-seq data and identification of cell clusters

The scRNA-seq data were processed using “Seurat” in the R project (27). Clusters with fewer than three cell counts were excluded. We retained cells with gene counts of more than 200 and less than 5000 and cells with less than 5% of mitochondrial genes. The data were normalized using the “NormalizeData” function to ensure the cells were comparable before feature extraction. The “FindVariableFeatures” function was used to find the top 2,000 highly variable genes. Then, we applied the “ScaleData” method to scale all the genes so that the same gene could be comparable in different samples. The “RunPCA” function was used to perform principal component analysis (PCA) on the 2,000 highly variable genes selected above, and the JackStraw plot identified the top 15 dimensions with p < 0.05. Cells were clustered using the “FindNeighbors” and “FindClusters” methods with a resolution of 0.4. Uniform Manifold Approximation and Projection (UMAP) is a new visualization and scalable dimensionality reduction algorithm. Compared to t-SNE, UMAP retains a more global structure, has superior runtime performance, and is more scalable (28). We used UMAP to visualize cell clusters. Genes with | log fold change | > 0.25 and at least 25% in the cluster were considered differentially expressed by the “FindAllMarkers” function. The cell clusters were annotated based on the known marker (29–32) and corrected using the CellMarker database (http://xteam.xbio.top/CellMarker). Finally, we used a threshold of | log2 fold change | > 1 and FDR < 0.05 to screen MRGs.

### 2.3 Prognostic signature construction

The 516 HNSCC patients’ data with complete survival and clinical information were divided into a training group (n = 322) and a validation group (n = 194) using the “caTools” method in a 7:3 ratio. In the training group, we used least absolute shrinkage and selection operator (LASSO) regression analysis to avoid overfitting and reduce genes. In order to assess the predictive significance of MRGs for OS in HNSCC patients, we conducted univariate Cox regression analysis and identified the prognostic genes with p < 0.05. Stepwise multivariate Cox regression analysis identified genes for the construction of prognostic signatures. MRGs-Riskscore were calculated for each patient based on regression coefficients and gene mRNA expression with the following formula:


$$\text{MRGs}-\text{Riskscore }=\;\sum_{n=1}^{\infty }\left[coefficient\left(n\right)*expression\left(n\right)\right]$$

Patients were classified into high- and low-risk groups based on the cut-off values calculated by the X-tile software. Kaplan-Meier survival curves were plotted using the “survival” function to visualize the difference in survival between the two risk groups. In addition, we depicted risk factor association plots to show the distribution of patients. The same regression coefficients and cut-off value were used for the validation group to test the risk stratification ability of the prognostic signature. The “ggpubr” method was used to generate boxplots to explore the relationship between MRGs-Riskscore and clinicopathological factors such as age, gender, stage, T-stage and N-stage.

### 2.4 Independent prognostic analysis and construction of nomogram

We performed an independent prognostic analysis of the 14-MRGs prognostic signature and clinicopathological factors. The Cox regression analysis was used to identify factors that had an independent effect on the patient’s prognosis. Characteristics with p < 0.05 in the multivariate Cox regression analysis were considered to be independent prognostic factors. In order to clarify the clinical value of the prognostic signature, we constructed a nomogram using the “rms” package, and calibration curves were used to evaluate the relationship between predicted and actual probabilities at 1-, 3-, and 5-year. The C-index and receiver operating characteristic (ROC) curves were drawn to assess the power of the nomogram and other independent prognostic factors in predicting the OS of patients.

### 2.5 Immune infiltration analysis and immune landscape

CIBERSORT is a deconvolution algorithm based on the principle of linear support vector regression, providing a gene expression signature matrix for 22 immune cells, which can be used to evaluate the relative abundance of different cell types in complex mixed tissue samples (33). We analyzed the infiltration of 22 immune cell types between high- and low-risk groups by CIBERSORT. In addition, we used six other immune infiltration algorithms to further reveal immune infiltration, such as CIBERSORT-ABS (33), XCELL (34), TIMER (35), QUANTISEQ (36), MCPCOUNTER (37), and EPIC (38). Since immune checkpoints played a crucial role in patient response to immunotherapy, we analyzed the expression levels of classical immune checkpoints in high- and low-risk groups. Correlation analysis among model genes and immune cells helped to understand cellular regulation by MRGs. The ESTIMATE algorithm (39) was used to assess the immune and stromal scores. Tumor immune dysfunction and exclusion (TIDE) was used to evaluate the likelihood of a patient’s response to immunotherapy (40).

### 2.6 Drug susceptibility analysis

Applying the “oncoPredict” package, we evaluated the half-maximal inhibitory concentration (IC50) of 198 drugs in the high- and low-risk groups to understand the difference in sensitivity between the two groups of patients. These included clinically used targeted and chemotherapeutic drugs such as cytarabine, gefitinib, acitretin, etc. The data were obtained from the GDSC database (https://www.cancerrxgene.org/).

### 2.7 Statistical analysis

All R packages were run by RStudio (version 4.3.1). The SPSS (version 25) was used for Cox regression and independent prognostic analyses. The X-tile software was used to calculate cut-off values. The Kaplan-Meier survival curves were compared using log-rank tests. The wilcoxon test was employed to compare the two groups. Correlation coefficients were calculated using spearman correlation analysis. A significance threshold of p <0.05 was established for determining significant differences.

## 3 Results

### 3.1 Identification of MRGs

**Figure 1:**
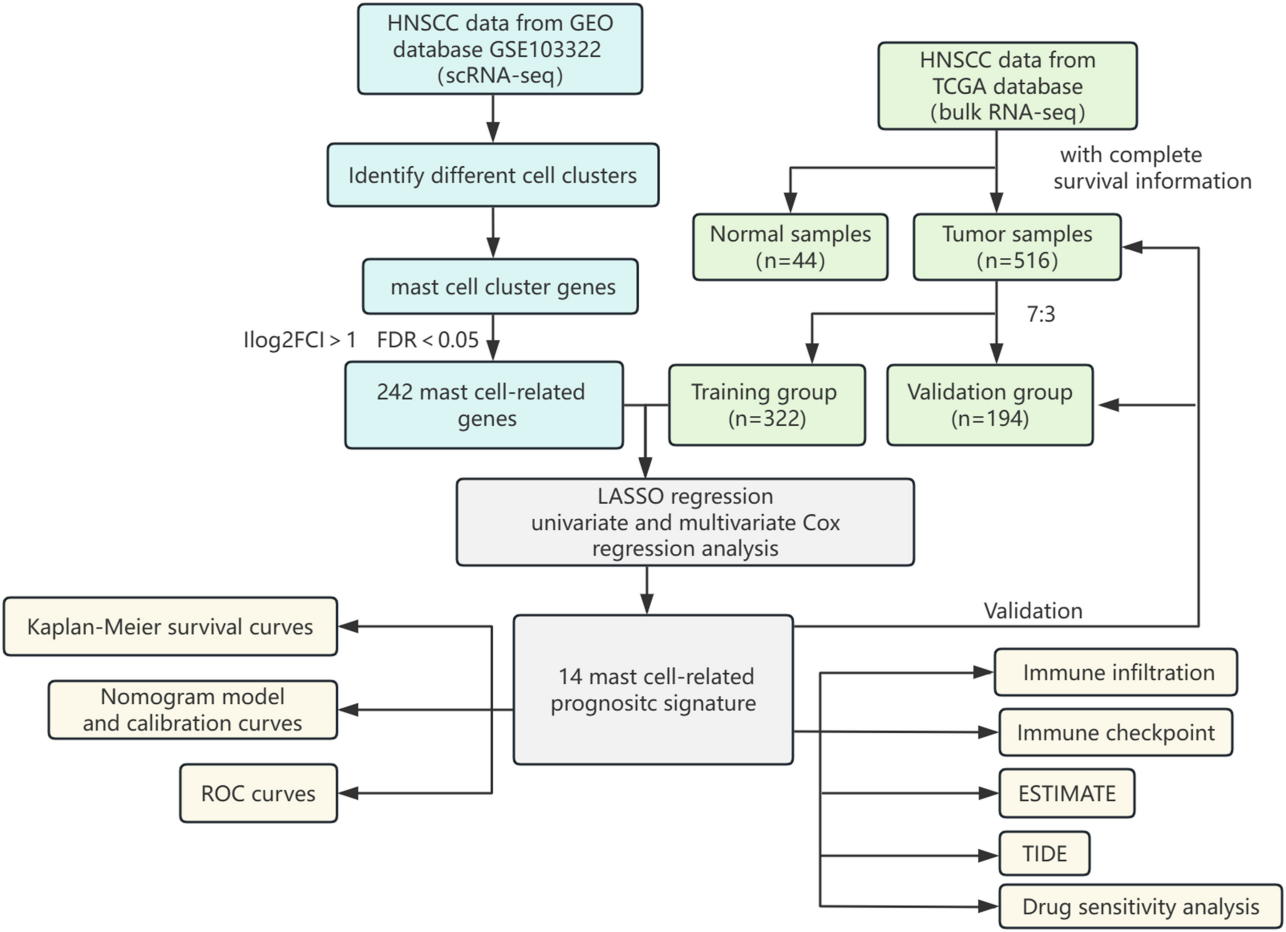
Flowchart of this study.

**Figure 2:**
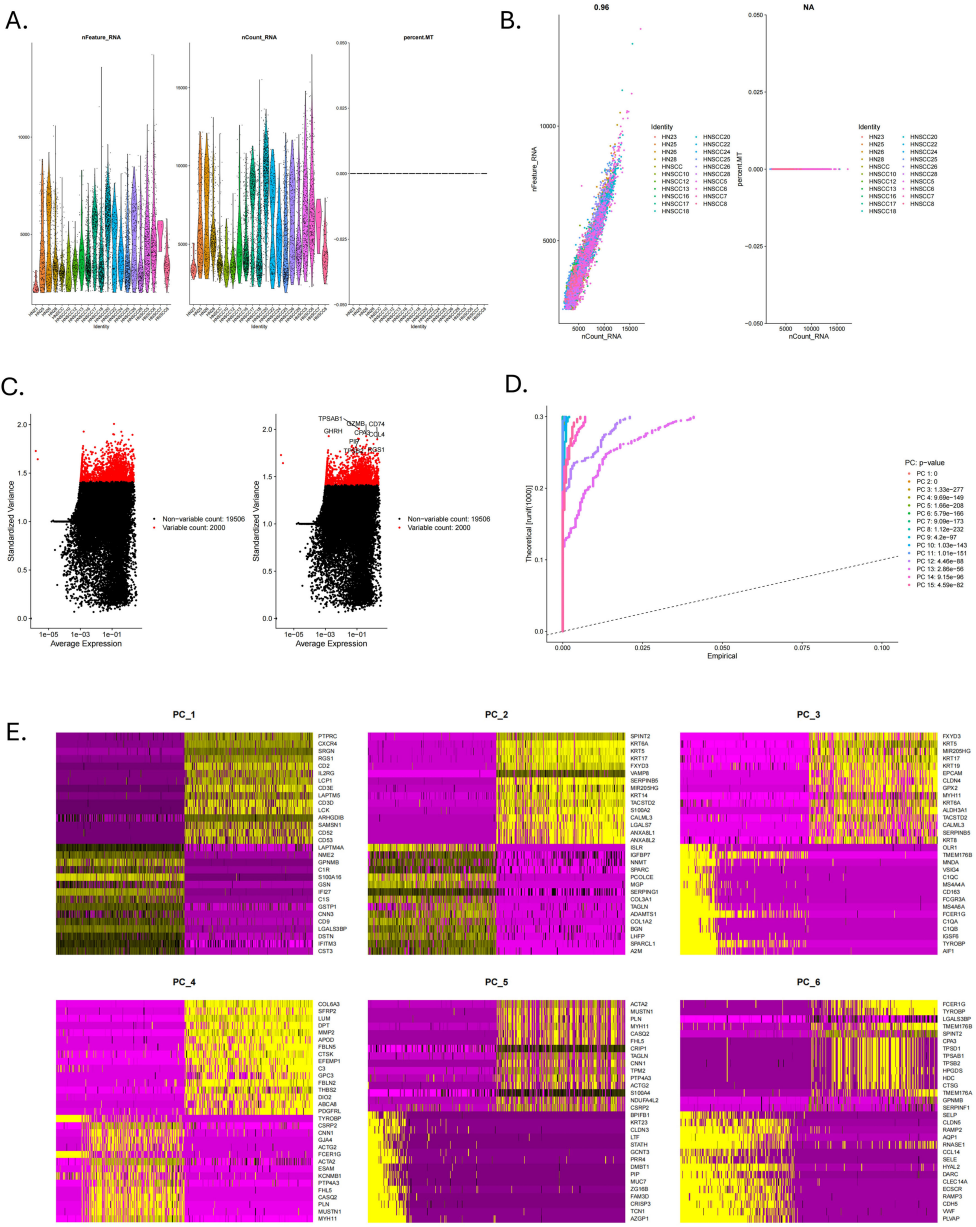
Quality control of scRNA-seq data. **(A)** The violin plots of RNA expression across different identities for features, counts, and mitochondrial percentage. **(B)** The scatter plot showed the correlation analysis between the sequencing depth and the percentage of expressed genes and mitochondria. **(C)** Top 2000 highly variable genes. **(D)** The Jackstraw plot illustrated principal component standard deviation, highlighting significant components. **(E)** Top 30 highly expressed genes of the first six PCAs.

**Figure 3:**
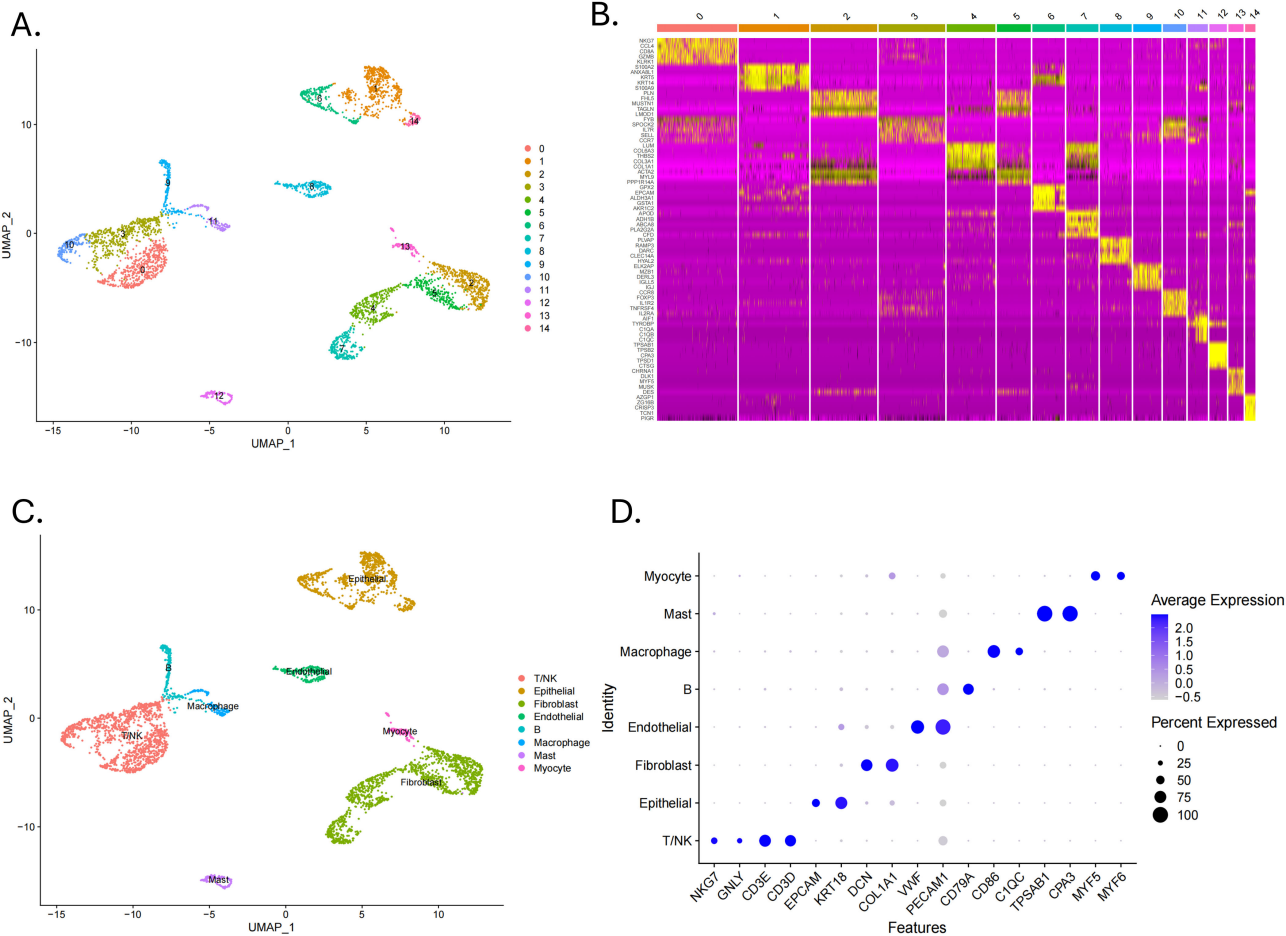
Identification of cell clusters by scRNA-seq technology. **(A)** A UMAP plot showed 15 clusters of data points in different colors, each representing a cluster from 0 to 14. **(B)** A heatmap displayed gene expression levels across clusters with a color scale. **(C)** A UMAP plot labelled clusters with cell identities such as T/NK, epithelial, and macrophage. **(D)** A dot plot showed average expression of various features across cell types, with dot size indicating percent expressed and color intensity indicating expression level.

The flowchart shows the major procedures of our study (**Figure 1**). After quality control of scRNA-seq data (**Figures 2A, B**), the expression matrix of genes from 4,033 cells was retained. We screened the top 2,000 highly variable genes and the top 15 PCA (**Figures 2C, D**). The top 30 highly expressed genes of the first six PCAs are shown in **Figure 2E**. All cells were divided into 15 cell clusters and annotated as T/NK cells, B cells, epithelial cells, fibroblasts, endothelial cells, macrophages, mast cells, and monocytes (**Figures 3A, B**). The cluster 12 was identified as mast cells (**Figure 3C**). Bubble plots demonstrated highly expressed genes in each cell cluster (**Figure 3D**). In cluster 12, 253 genes were screened according to the thresholds of **|** log2 fold change **|** > 1 and FDR < 0.05. Mapped to the TCGA database gene list, 242 genes were retained for subsequent analysis.

### 3.2 14-MRGs prognostic signature construction and validation

**Figure 4:**
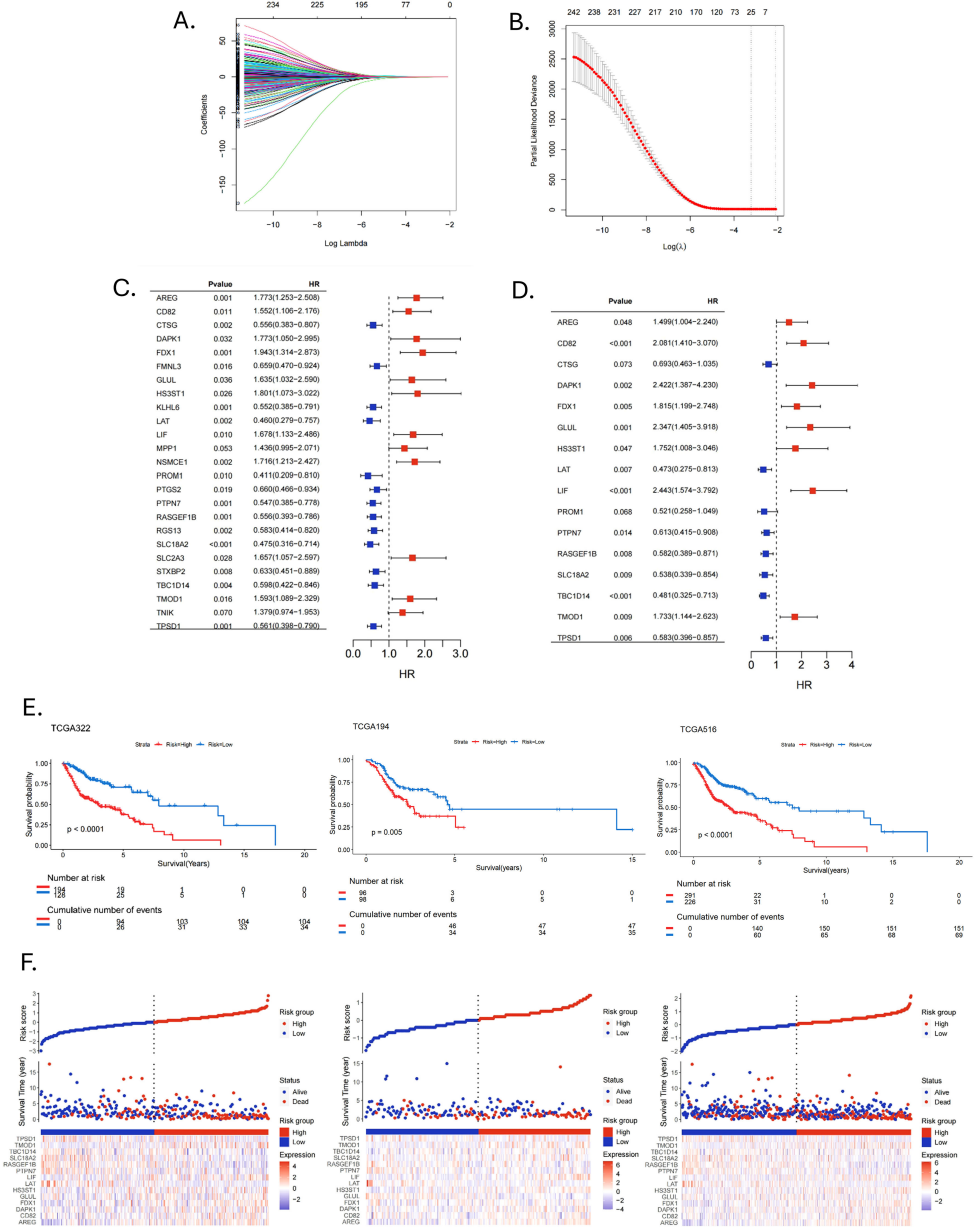
Construction and validation of prognostic signature. **(A, B)** LASSO regression analysis identified signature genes. **(C)** Univariate Cox regression analysis screened prognostic genes (p < 0.05). **(D)** Multivariate Cox regression analysis screened prognostic genes (p < 0.05). **(E)** Kaplan-Meier survival curves assessed the ability to stratify patients by prognostic signatures in the training, validation and entire cohorts. **(F)** Risk factor correlation plots in the training, validation and entire cohorts.

The PCA plot of batch effect removal in the TCGA database is shown in **Supplementary Figure 1**. The data of 516 HNSCC patients in the TCGA were divided into training and validation groups in a ratio of 7:3. **Table 1** demonstrates the subgroups and basic characteristics. We performed LASSO regression to screen out 25 OS-related MRGs from 242 genes (**Figures 4A, B**). By univariate Cox regression analysis, we obtained 23 MRGs associated with the OS of patients (**Figure 4C**, p < 0.05). The multivariate Cox regression analysis further identified 14 OS-related MRGs to construct a prognostic signature, including AREG, CD82, DAPK1, FDX1, GLUL, HS3ST1, LAT, LIF, PTPN7, RASGEF1B, SLC18A2, TBC1D14, TMOD1 and TPSD1 (**Figure 4D**, p < 0.05). **Table 2** shows the information of each prognostic gene. Kaplan-Meier survival curves of each MRG are shown in **Supplementary Figure 2**. MRGs-Riskscore were calculated for each patient based on regression coefficients and gene mRNA expression: MRGs-Riskscore = 0.405 * exp (AREG) + 0.733 * exp (CD82) + 0.885 * exp (DAPK1) + 0.596 * exp (FDX1) + 0.853 * exp (GLUL) + 0.561 * exp (HS3ST1) - 0.749 * exp (LAT) + 0.893 * exp (LIF) - 0.489 * exp (PTPN7) - 0.542 * exp (RASGEF1B) - 0.620 * exp (SLC18A2) - 0.731 * exp (TBC1D14) + 0.550 * exp (TMOD1) -0.540 * exp (TPSD1). Patients were categorized into high- and low-risk groups based on the optimal cut-off value (15.94). Survival analysis showed that patients in the low-risk group had longer OS compared to the high-risk group. In the validation group and the entire cohort, survival analysis showed the same trends as in the training group (**Figure 4E**). Risk factor correlation plots showed the distribution of patients (**Figure 4F**).

**Table 1:** Clinicopathologic information and subgroups of HNSCC patients.

Patient characteristics	Training group		Validation group		Entire cohort		P value
	n=322	%	n=194	%	n=516	%	
Age							0.113
≤74	287	89.13	159	81.96	446	86.43	
>74	35	10.87	35	18.04	70	13.57	
Gender							0.960
Female	84	26.08	51	26.29	135	26.16	
Male	238	73.92	143	73.71	381	73.84	
Stage							0.110
I	16	4.97	11	5.67	27	5.23	
II	47	14.60	35	18.04	82	15.89	
III	53	16.46	40	20.62	93	18.02	
IV	206	63.97	108	55.67	314	60.86	
T							0.390
T1	31	9.63	20	10.31	51	9.88	
T2	95	29.50	61	31.44	156	30.23	
T3	73	22.67	48	24.74	121	23.45	
T4	123	38.20	65	33.51	188	36.44	
N							0.513
N0	135	41.93	86	44.33	221	42.83	
N1	49	15.22	31	15.98	80	15.50	
N2	131	40.68	73	37.63	204	39.54	
N3	7	2.17	4	2.06	11	2.13	
Vital status							0.896
Alive	184	47.14	112	57.73	296	57.36	
Dead	138	42.86	82	42.27	220	42.64	

**Table 2:** Fourteen MRGs associated with prognosis in HNSCC patients.

Gene	Coefficient	HR(95%CI)	P value
AREG	0.405	1.499 (1.004-2.240)	0.048
CD82	0.733	2.081 (1.410-3.070)	0.000
DAPK1	0.885	2.422 (1.387-4.230)	0.002
FDX1	0.596	1.815 (1.199-2.748)	0.005
GLUL	0.853	2.347 (1.405-3.918)	0.001
HS3ST1	0.561	1.752 (1.008-3.046)	0.047
LAT	-0.749	0.473 (0.275-0.813)	0.007
LIF	0.893	2.443 (1.574-3.792)	0.000
PTPN7	-0.489	0.613 (0.415-0.908)	0.014
RASGEF1B	-0.542	0.582 (0.389-0.871)	0.008
SLC18A2	-0.620	0.538 (0.339-0.854)	0.009
TBC1D14	-0.731	0.481 (0.325-0.713)	0.000
TMOD1	0.550	1.733 (1.144-2.623)	0.009
TPSD1	-0.540	0.583 (0.396-0.857)	0.006

**Figure 5:**
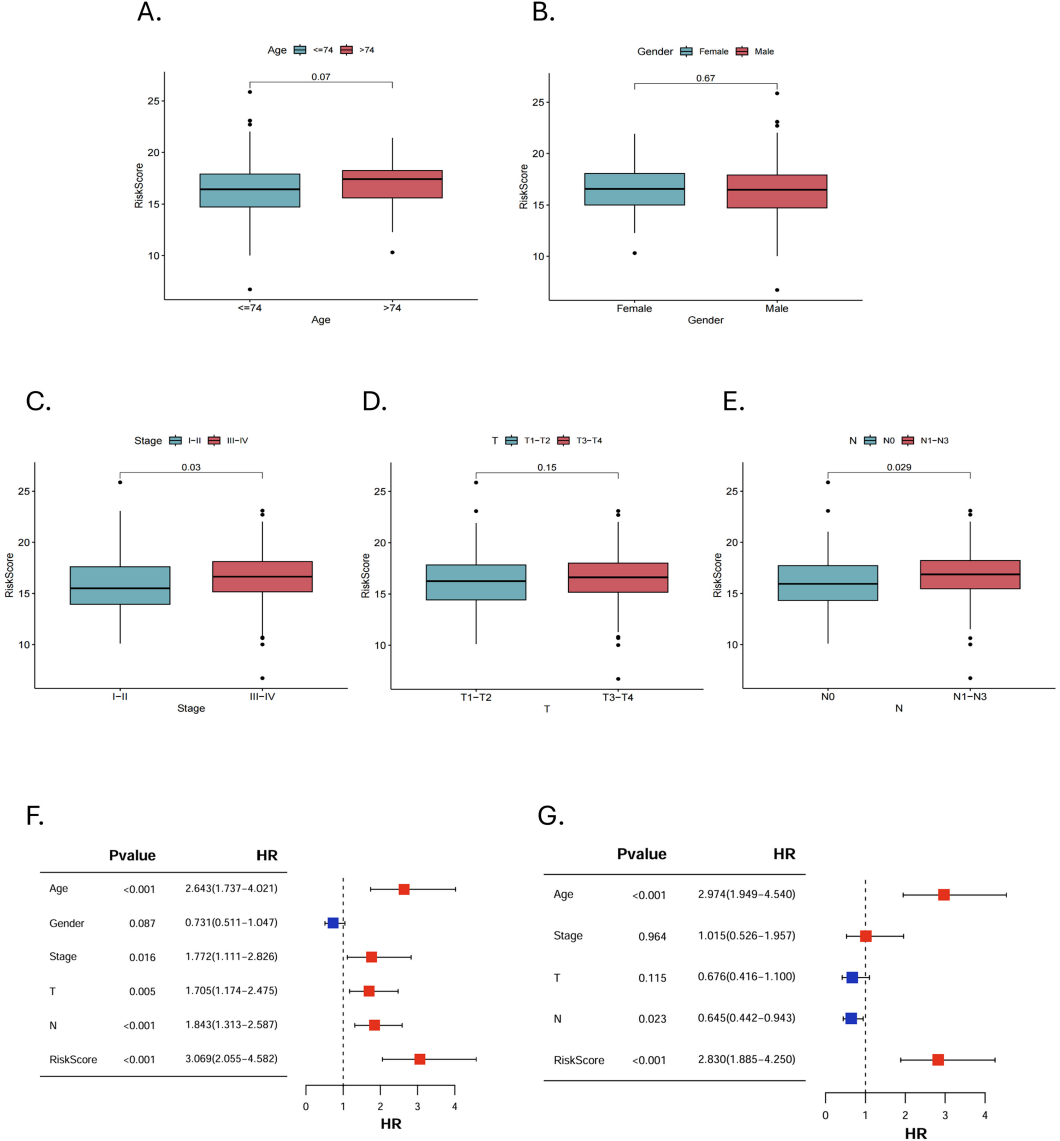
Clinical pathological risk stratification and independent prognostic analysis. Stratified risk of patients in different clinicopathological subgroups, including **(A)** age, **(B)** gender, **(C)** stage, **(D)** T stage and **(E)** N stage. **(F, G)** Univariate and multivariate Cox regression analyses to select features with independent prognostic ability.

We further analyzed the risk stratification of clinicopathological factors. There was no significant difference between MRGs-Riskscore and clinical characteristics, including age, gender and stage T (**Figures 5A, B, D**). Patients with advanced stages had a higher MRGs-Riskscore than stage I-II (**Figure 5C**, p = 0.03). In addition, there was a higher MRGs-Riskscore in the N1-N3 stage compared to the N0 stage (**Figure 5E**, p = 0.029). We performed an independent prognostic analysis of MRGs-Riskscore and clinicopathological factors. The results of univariate Cox analysis showed that age, stage, T stage, N stage, and MRGs-Riskscore were correlated with OS (**Figure 5F**, p < 0.05). The results of stepwise multivariate Cox regression analysis showed that age, N stage, and MRGs-Riskscore were independent predictors of HNSCC patients (**Figure 5G**, p < 0.05).

### 3.3 Nomogram construction and validation

**Figure 6:**
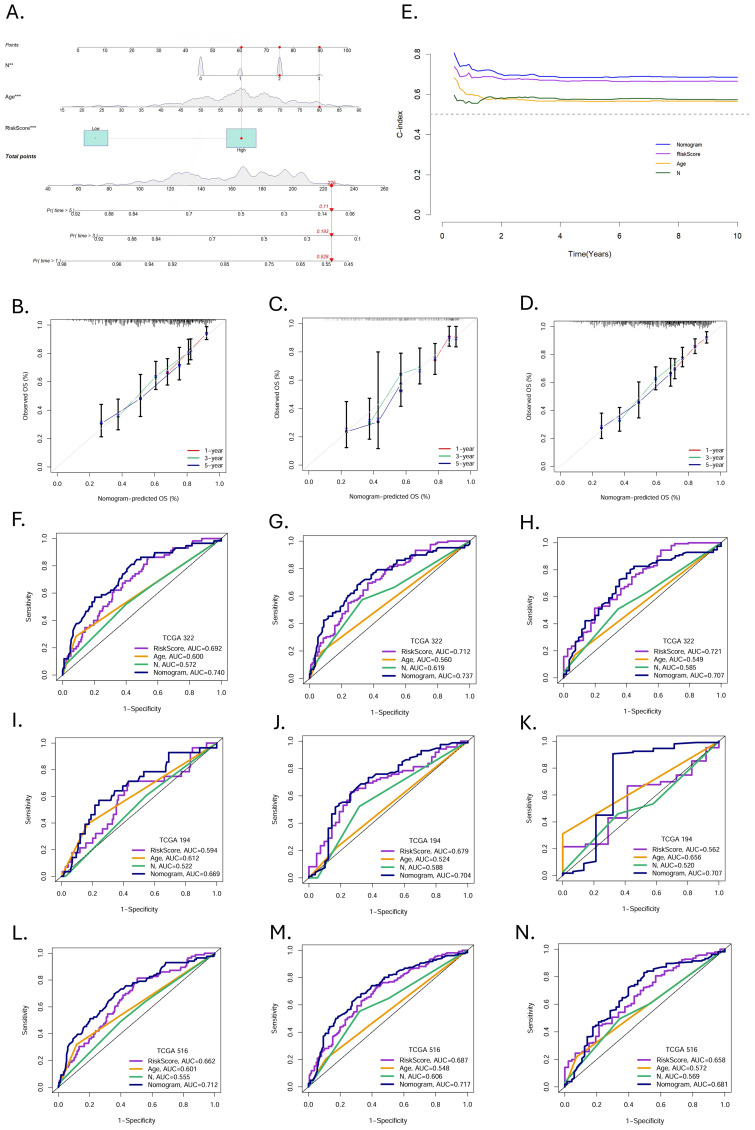
Construction and validation of nomogram. **(A)** Nomogram prediction models were developed to forecast the OS of HNSCC patients at 1-, 3-, and 5year (*p < 0.05, **p < 0.01, ***p < 0.001). **(B-D)** Calibration curves at 1-, 3-, and 5-year in the training, validation, and entire cohorts. **(E)** The C-index over ten years for different predictors. ROC curves evaluated the predictive performance of prognostic factors at 1-, 3-, and 5 years in the **(F-H)** training, **(I-K)** validation, and **(L-N)** entire cohort.

In order to clarify the value of clinical application, we constructed a nomogram based on independent predictors (**Figure 6A**). Summing the corresponding scores of each indicator, we could predict the survival rate of patients at 1-, 3-, and 5-year.

The calibration curves demonstrated excellent consistency between the nomogram predicted probabilities and the actual observations at 1-, 3-, and 5-year OS. The same performance was observed in the validation group and the entire cohort (**Figures 6B–D**). The C-indices of nomogram, MRGs-Riskscore, age, and N stage were 0.69565382, 0.67153782, 0.57232907, and 0.57672068, respectively (**Figure 6E**). In the training group, the AUC of the nomogram at 1-, 3-, and 5-year were 0.740, 0.737, and 0.707 (**Figures 6F–H**). In the validation group, the AUC at 1-, 3-, and 5-year were 0.669, 0.704, and 0.707 (**Figures 6I-K**), and in the entire cohort, the AUC at 1-, 3-, and 5-year were 0.712, 0.717, and 0.681 (**Figures 6L-N**). Nomogram have a more favorable predictive performance than other clinicopathological factors.

### 3.4 Immune cells infiltration and immune landscape in HNSCC

**Figure 7:**
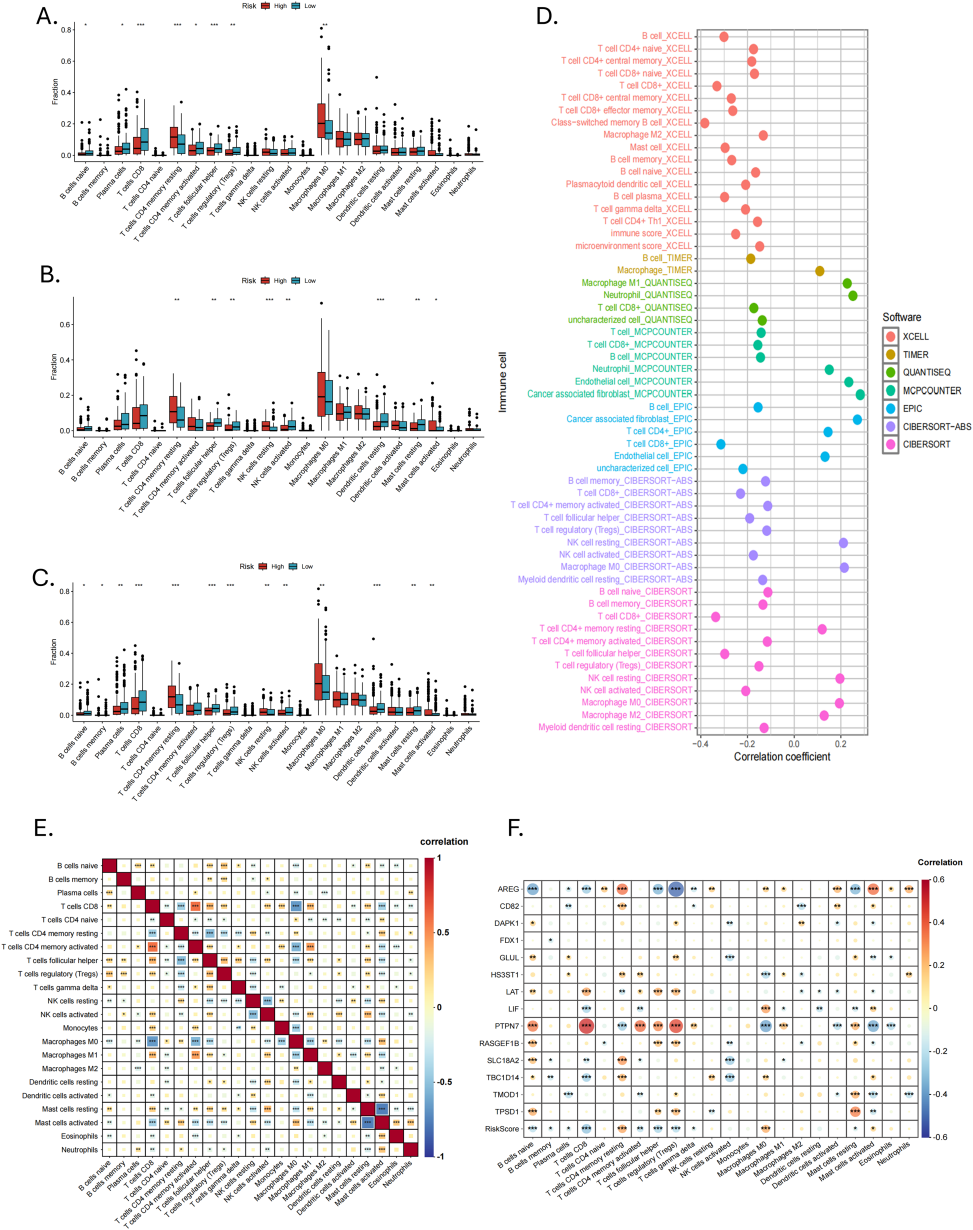
Immune infiltration and correlation analysis. The infiltration of 22 immune cell types between high- and low-risk groups in the **(A)** training, **(B)** validation and **(C)** entire cohorts. **(D)** Seven immune infiltration algorithms. **(E)** Correlation analysis of 22 immune cell types (*P < 0.05, **P < 0.01, ***P < 0.001). **(F)** Correlation analysis of 14 MRGs and 22 types of immune cells (*P < 0.05, **P < 0.01, ***P < 0.001).

In the TME, intercellular communication is closely related to tumor progression. We used CIBERSORT to reveal the immune infiltration of HNSCC in different risk groups. As shown in **Figures 7A, B**, there were eight types of immune cells separately, with significant differences between the high- and low-risk groups in the training and validation groups. At the same time, in the entire cohort, there were 13 types (**Figure 7C**). Immune cells with significant infiltration differences in all three groups contained resting CD4+ memory T cells, follicular helper T cells (Tfhs) and regulatory T cells (Tregs). In addition, we found that in the validation group and the entire cohort, resting and active mast cells showed opposite trends in infiltration between the two risk groups.

We used six other immune infiltration algorithms and found that the low-risk group had a richer infiltration of anti-tumor immune cells, such as CD8+ T cells, CD4+ T cells, active NK cells and B cells (**Figure 7D**). The high-risk group mainly consisted of cancer-associated fibroblasts (CAFs), neutrophils, endothelial cells, and some immune cells in a resting state. Correlation analysis between 22 immune cell types showed that CD8+ T cells, CD4+ memory T cells, Tfhs, Tregs, NK cells, macrophages, eosinophils, and neutrophils were correlated with mast cells. This correlation may be related to mast cells in an active or resting state (**Figure 7E**). Correlation analysis of the 14 MRGs with immune cells showed a strong correlation of AREG and PTPN7 with a wide range of immune cells (**Figure 7F**).

**Figure 8:**
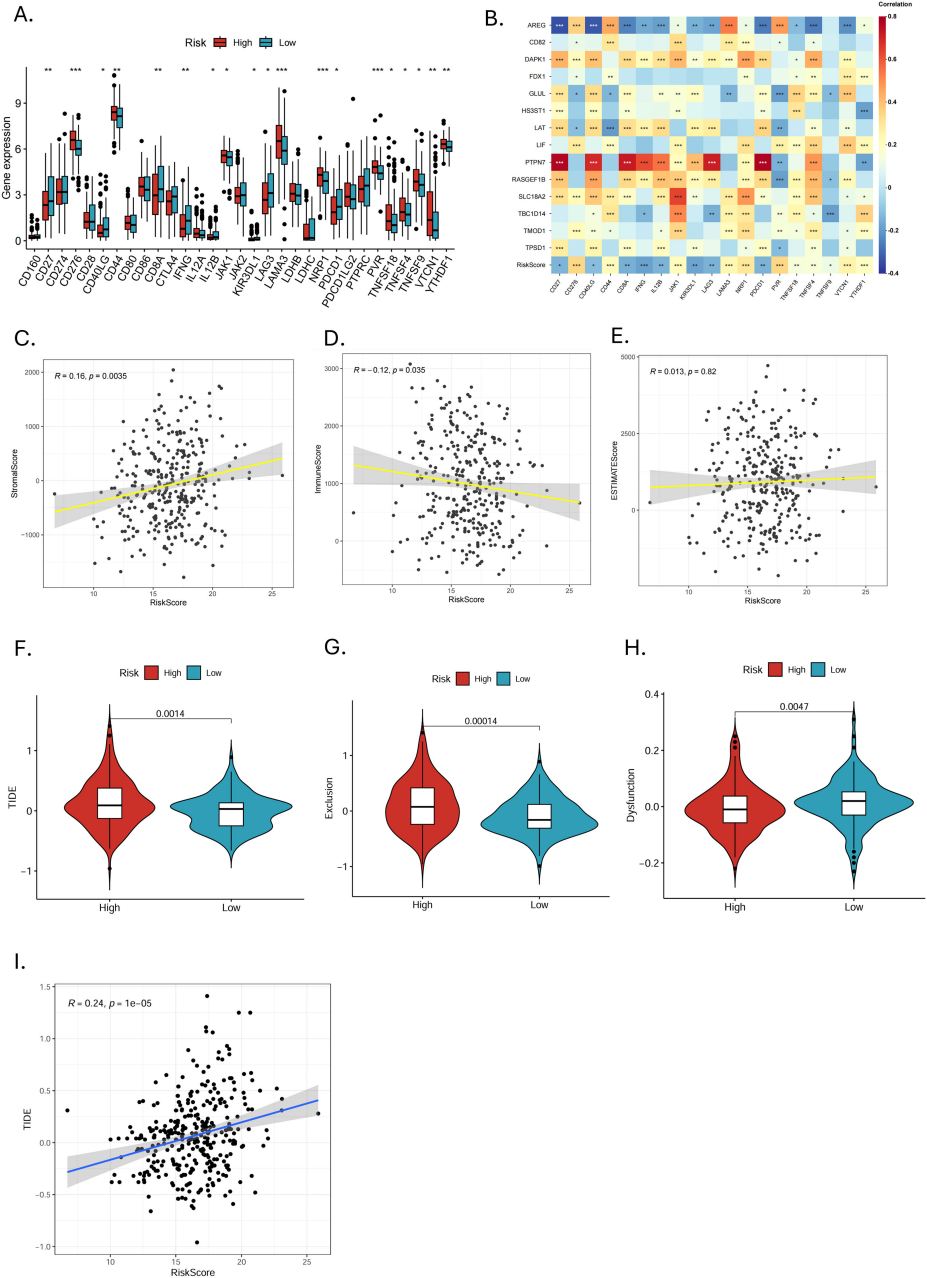
Immune checkpoint expression and prediction of immunotherapy response. **(A)** Differences in immune checkpoint expression between high- and low-risk groups (*p < 0.05, **p < 0.01, ***p < 0.001). **(B)** Correlation analysis of 14 MRGs and differentially expressed immune checkpoints (*p < 0.05, **p < 0.01, ***p < 0.001). **(C-E)** Correlation analysis of MRGs-RiskScore with stromal score, immune score, and ESTIMATE score. **(F-H)** Analysis of TIDE, exclusion score, and dysfunction score in high- and low-risk groups. **(I)** Scatter plot of correlation between MRGs-RiskScore and TIDE.

The expression of 31 immune checkpoints between high- and low-risk groups was shown in the box plot (**Figure 8A**). Highly expressed in the high-risk group were CD276, CD44, JAK1, KIR3DL1, LAMA3, NRP1, PVR, TNFSF18, TNFSF4, TNFSF9, VTCN1, and YTHDF1, while in the low-risk group, CD27, CD40LG, CD8A, IFNG, IL12B, LAG3, and PDCD1 showed high expression. In the correlation analysis (**Figure 8B**), AREG, PTPN7, RASGEF1B, LAT and GLUL were highly correlated with several immune checkpoints, especially AREG and PTPN7, suggesting that these genes may mainly influence the expression of immune checkpoints. The ESTIMATE analysis revealed that MRGs-Riskscore was positively correlated with stromal scores and negatively correlated with immune scores (**Figure 8C**). What is more, the low-risk group had lower TIDE scores (**Figure 8D**, p = 0.0014) and immune exclusion scores (**Figure 8E**, p = 0.00014), as well as higher T-cell dysfunction scores (**Figure 8F**, p = 0.0047). The MRGs-Riskscore was positively correlated with the TIDE score (**Figure 8G**), suggesting that low-risk patients are more likely to benefit from immunotherapy.

### 3.5 Drug susceptibility analysis in high- and low-risk groups

**Figure 9:**
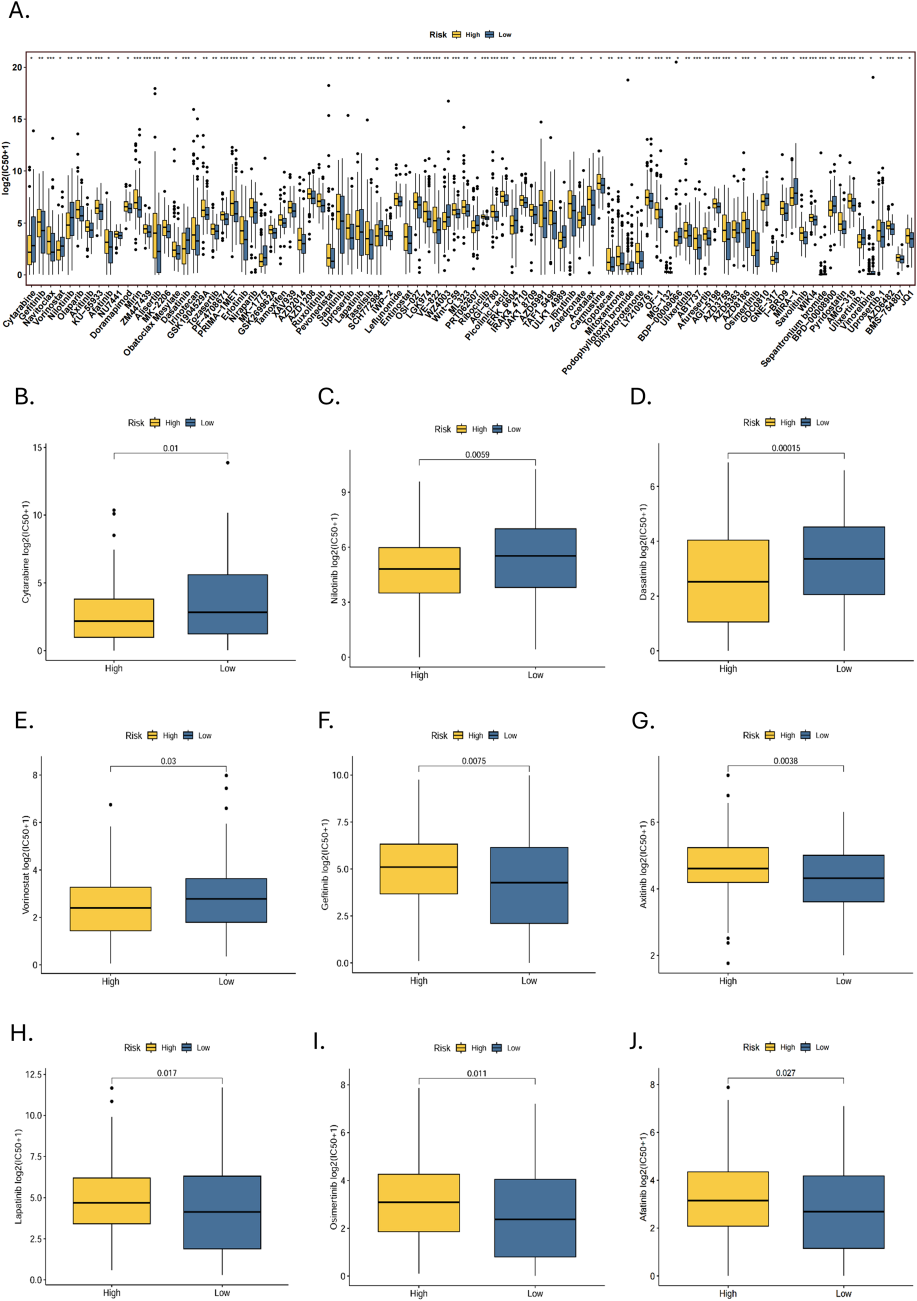
Drug susceptibility analysis. **(A)** A statistically significant difference in IC50 between high- and low-risk groups for 92 drugs (*p < 0.05, **p < 0.01, ***p < 0.001). Targeted drugs that are sensitive to the **(B-E)** high-risk group and **(F-J)** low-risk group.

Sensitivity analyses of 198 drugs in the high- and low-risk groups showed that 92 drugs had statistically significant differences in IC50 (**Figure 9A**). Of these, The high-risk group was more sensitive to 18 drugs, while the low-risk group was sensitive to 74 drugs. Among the commonly used targeted and chemotherapeutic agents, the high-risk group was sensitive to cytarabine, nilotinib, dasatinib and vorinostat (**Figures 8B–E**). Those sensitive in the low-risk group were gefitinib, axitinib, lapatinib, osimertinib and afatinib (**Figures 8F-J**).

## 4 Discussion

HNSCC is a highly heterogeneous and aggressive tumor. Currently, ICIs are a major treatment for HNSCC. However, the therapeutic effect is not optimistic. Mast cells are one of the widespread immune cells in the human body, and their tumor-promoting and anti-tumor-enhancing immunity exerted in the TME in various ways demonstrates their potential as a target for tumor immunotherapy. In this study, we attempted to construct a prognostic model for HNSCC based on MRGs to predict the OS of patients and reveal the immune infiltration and landscape of HNSCC to screen patients who might benefit from immunotherapy.

In order to have a deeper understanding of each gene, we investigated the role of 14-MRGs in cancers. In our study, survival analysis showed that LAT, PTPN7, RASGEF1B, SLC18A2, TBC1D14, and TPSD1 were protective factors and associated with longer OS, while higher expressions of AREG, CD82, DAPK1, FDX1, GLUL, HS3ST1, LIF, and TMOD1 were associated with shorter OS. It has been reported that AREG, CD82, LIF, TBC1D14, TPSD1, DAPK1, FDX1, GLUL, and HS3ST1 affected the survival prognosis of HNSCC patients. AREG encodes proteins that are members of the epidermal growth factor (EGF) family and regulate proliferation, apoptosis and migration in different cell types. High expression of AREG was correlated with a poorer OS in HPV-associated HNSCC (41). Several studies have also shown that AREG is a poor prognostic factor in HNSCC (42–44). In addition, mast cells are a potential major source of AREG (45). LIF is a pleiotropic factor that has been shown to be associated with poor tumor prognosis in a wide range of tumors (46–48). Dayson et al. suggested that radiotherapy may have enhanced the immunosuppression of HNSCC, induced significant LIF gene signaling, and reduced the role of cytotoxic lymphocytes (49). LAT, PTPN7, RASGEF1B, SLC18A2, and TMOD1 have not been reported in HNSCC. LAT is a connexin for T-cell activation. Phosphorylated LAT can bind to various signaling proteins to form a multiprotein complex, which plays an essential role in T-cell activation and regulation and promotes the cytotoxicity of CD8+ cytotoxic T lymphocytes (CTLs) (50). PTPN7 is involved in immune infiltration and is strongly correlated with immunothermal tumors in breast cancer (51). In digestive tract cancer (52), bladder cancer (53) and glioma (54), PTPN7 can serves as a predictive tumor biomarker. TMOD1, a member of the encoded pro-regulatory protein family, plays a vital role in regulating the organization of actin filaments. High expression of TMOD1 has been confirmed to correlate with tumor growth and enhanced lymph node metastasis (55, 56).

These findings are consistent with our study. It is worth noting that several studies have shown that PTPN7 is an unfavorable prognostic factor in tumors, contrary to our results, which suggest that the role played by PTPN7 in tumors and the mechanisms affecting prognosis are complex. We need further *in vivo* and *in vitro* experiments to clarify the function of PTPN7 in HNSCC. In correlation analyses, we found that AREG and PTPN7 are highly associated with a wide range of immune cells and immune checkpoints, suggesting that they may be involved at the molecular level in regulating cellular interactions as well as immune checkpoint expression in the TME of HNSCC.

Immune cells in TME play a vital role in the dynamic progression of HNSCC (57). We found that more immune cells were enriched in the low-risk group through immune infiltration analysis. Tregs, Tfhs, and resting CD4+ memory T cells significantly different between high- and low-risk groups in three sets. In tumor development, the surrounding microenvironment produces cross-linkages and gradually creates conditions conducive to tumor growth and invasion. This process is achieved by depleting anti-tumor immune cells (mainly T cells) to promote immunosuppression. For example, PD-L1 binding to PD-1 leads to T cell dysfunction by reducing T cell receptor (TCR) signaling and promoting differentiation into Tregs, making immune escape easy (58, 59). Tregs can produce the immunosuppressive cytokines IL-10 and TGF-β to deplete IL-2, constituting the CD3+CD4+ subpopulation to suppress the activity of effector T cells and effective anti-tumor immune response (60). Our study showed a significant infiltration difference of CD8+ T cells between high- and low-risk groups. Thus, CD8+ T cells may be depleted by Tregs. Depletion of CD8+ T cells and other essential components of anti-tumor immunity may be the main reason for the limited efficacy of long-term immunotherapy in humans (58). However, under specific circumstances, Tregs can inhibit the malignant transformation of tumors (61). Some studies have reported that high levels of Tregs in HNSCC are associated with longer recurrence-free survival (RFS) and OS, which may be closely related to high levels of CD4+CD25+Foxp3+Tregs in TILs (60). Several other studies have demonstrated that high levels of Tregs infiltration can improve the OS of HNSCC patients. What is more, Foxp3+Treg is considered a good independent prognostic factor for HNSCC (62–64). Thus, specific phenotypes of immune cells determine their function in tumors. Tfhs play different roles in Tfh cell-derived tumors, B-cell lymphomas, and solid organ tumors. In HPV+HNSCC, CD4+ Tfh cells can assist in efficiently activating TIL-B, thereby enhancing anti-tumor immunity (65). The differentiation of resting CD4+ memory T cells into Th cells with different phenotypes may provide new directions for the mechanism and immunotherapy of HNSCC (66).

In our study, there was no significant difference in mast cell infiltration between the high-risk and low-risk groups in the training group. This may be due to the larger tumor heterogeneity among the patients in this group, which reduces the statistical significance. Secondly, the sample size of this group may not be sufficient to detect significant infiltration differences. Nonetheless, mast cells in different states in the training group still show an infiltration trend, activated mast cells infiltrate more in the high-risk group, while resting mast cells are more abundant in the low-risk group. The results of multiple immune infiltration algorithms demonstrated that the low-risk group generally had a richer infiltration of anti-tumor immune cells, such as CD8+ T cells, CD4+ T cells, active NK cells, B cells, and so on. The ESTIMATE showed that the MRGs-Riskscore were negatively correlated with the immune scores. Higher immune scores are usually associated with a favorable prognosis (67, 68). It illustrated why the OS and prognosis of patients in the low-risk group were better than those in the high-risk group. In addition, the lower TIDE score and immune exclusion score in the low-risk group suggest that patients in this group are less likely to experience immune escape and have a greater chance of benefiting from immunotherapy. The higher T-cell dysfunction score in the low-risk group may be related to suppressed T-cell function due to high Tregs infiltration.

After activation in the tumor microenvironment (TME), mast cells can release various mediators through degranulation, participating in physiological and pathological activities. For example, they can release molecules that promote tumor angiogenesis, such as vascular endothelial growth factor-A (VEGF-A), transforming growth factor-β (TGF-β), heparin, interleukin-8 (IL-8), matrix metalloproteinase-9 (MMP-9), trypsin-like proteases, and chymotrypsin (69). They can also release vascular endothelial growth factor-C (VEGF-C) and vascular endothelial growth factor-D (VEGF-D) to promote lymphangiogenesis, enhancing the tumor’s distal infiltration ability (70, 71). In addition, mast cells can secrete different cytokines to recruit CD8+ T cells or immune cells with suppressive phenotypes, such as tumor-associated macrophages (TAMs) and myeloid-derived suppressor cells (MDSCs), to the tumor periphery, achieving anti-tumor immunity or enhancing immune suppression (72, 73). In our study, active mast cells and resting mast cells were correlated and showed opposite trends with a variety of immune cells such as T cells, B cells, NK cells and macrophages, indicating that mast cells in certain states may release factors to communicate with other immune cells, thereby promoting or inhibiting tumor progression. Phenotypic and functional characterization of mast cells in different states is essential to advance immunotherapy for HNSCC.

ICIs treatment is one of the main treatment methods for HNSCC. It protects T-cell function by blocking the interaction between inhibitory receptors and their ligands, thereby reducing immune escape (74). Nivolumab and pembrolizumab are currently commonly used anti-PD-1 drugs. However, the monotherapy or combination application of ICIs from other pathways may provide new directions for the immunotherapy of HNSCC. Wang et al.’s research shows that CD276 is highly expressed in cancer stem cells (CSCs). During the occurrence, progression, and metastasis of HNSCC, CSCs evade immune system surveillance by upregulating the expression of CD276. Studies have shown that targeting CD276 can significantly enhance the CD8+T cell-mediated clearance of CSCs and inhibit the metastasis of HNSCC (75). This discovery highlights the unique potential of CD276 in HNSCC immunotherapy, making it a promising therapeutic target. To study the impact of CD44+ tumors on tumor angiogenesis, Nils Ludwig et al.’s research used tissue microarray technology combined with immunohistochemical methods to analyze the correlation between CD44 expression and microvessel density in HNSCC samples. The results showed that CD44+ tumor cells can secrete pro-angiogenic factors, thereby promoting angiogenesis in HNSCC. Based on this finding, CD44+ may serve as a potential marker for tumor angiogenesis and become an important target for anti-angiogenic therapy (76). The epidermal growth factor receptor (EGFR) is a member of the tyrosine kinase family, which can mediate cell proliferation and signal transduction after binding with EGF. EGFR is overexpressed in 80%-90% of HNSCC cases and is associated with poor OS and progression-free survival (PFS) (77, 78). Therefore, EGFR-targeted drugs such as cetuximab have become one of the treatment options for chemotherapy-resistant patients with HNSCC. Bonner et al.’s research explored whether dual inhibition using cetuximab and JAK-STAT-3 inhibition (JAK1i) could enhance the effect of cetuximab. The results showed that the antiproliferative effect of cetuximab was significantly enhanced after adding JAK1i, with greater radiosensitization (79). LAG3 is mainly significantly expressed in activated CD4+ T cells, CD8+ T cells and Tregs (80–82). LAG3 can rapidly translocate to the cell surface after activation, and this dynamic cell surface localization mechanism may be closely related to regulating its immunosuppressive function (83). Relatlimab is the first monoclonal antibody targeting LAG3 to be approved by the FDA. In phase I and I/IIa clinical trials for HNSCC, preliminary results indicate that relatlimab, whether used as monotherapy or in combination with anti-PD-1, exhibits good tolerability, efficacy, and controllable toxicity characteristics. These findings suggest that the dual immunotherapy combination of relatlimab and PD-1 has the potential to become a strategy to overcome immunotherapy resistance, providing new ideas for the immunotherapy of HNSCC (84). In our study, there were significant differences in the expression levels of 19 immune checkpoints between the high- and low-risk groups, distinguished by the 14-MRGs prognostic signature. Selectively applying ICIs therapy to patients in different risk groups may help improve efficacy and reduce the risk of drug resistance.

We must acknowledge some limitations in this study. This study is based on retrospective data from public databases. Although we have assessed the robustness of the model through internal validation by grouping and stratified analysis in the TCGA dataset, due to the excessive number of model genes and the lack of survival data of data sets, no suitable data sets have been found in GEO or other databases for external validation, which is a deficiency of this study. We will continue to seek appropriate independent datasets to complete external validation. Future research should include prospective validation with multicenter samples to enhance the current model’s general applicability and improve its clinical feasibility.

## 5 Conclusion

In this study, we applied scRNA-seq technology to identify MRGs, constructed a 14-MRGs prognostic model for HNSCC, and revealed the immune infiltration and landscape. These results suggest that patients in the low-risk group have more prolonged survival and richer immune cell infiltration and are more likely to benefit from immunotherapy, maybe provide appropriate and effective treatment options for HNSCC patients.

## Data Availability

The original contributions presented in the study are included in the article/**Supplementary Material**. Further inquiries can be directed to the corresponding authors.
