# Supplementary material for: A novel mast cell marker gene-related prognostic signature to predict prognosis and reveal the immune landscape in head and neck squamous cell carcinoma
Source: Front Immunol. 2025 Jul 9;16:1538641. doi: 10.3389/fimmu.2025.1538641 (PMC12283675; doi:10.3389/fimmu.2025.1538641)
Supplement: Supplementary file 1 [file DataSheet1.docx]

Supplementary Material

# Supplementary Figures


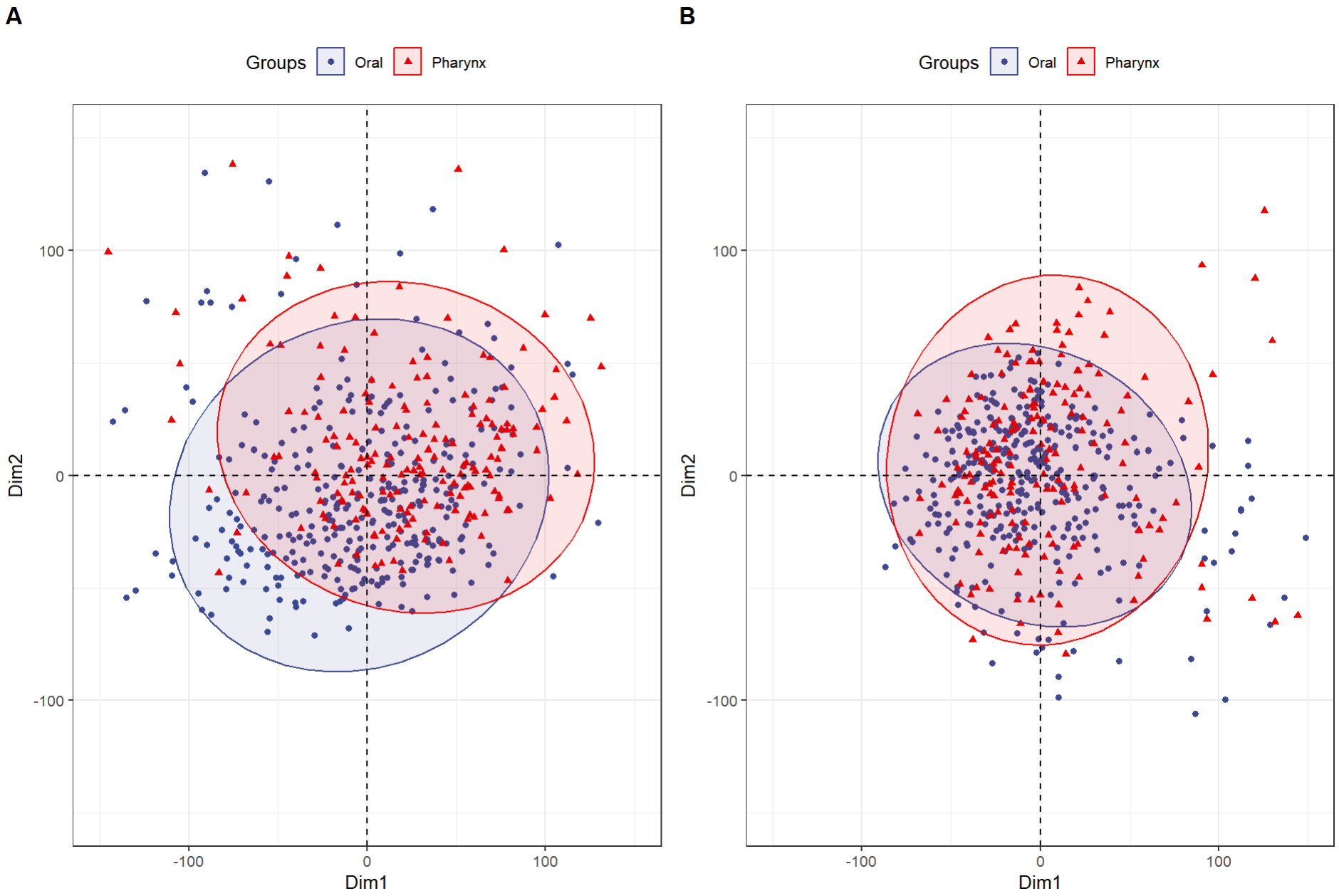


**Supplementary Figure 1.** **(A)** The PCA plots before batch removal. **(B)** The PCA plots after batch removal.


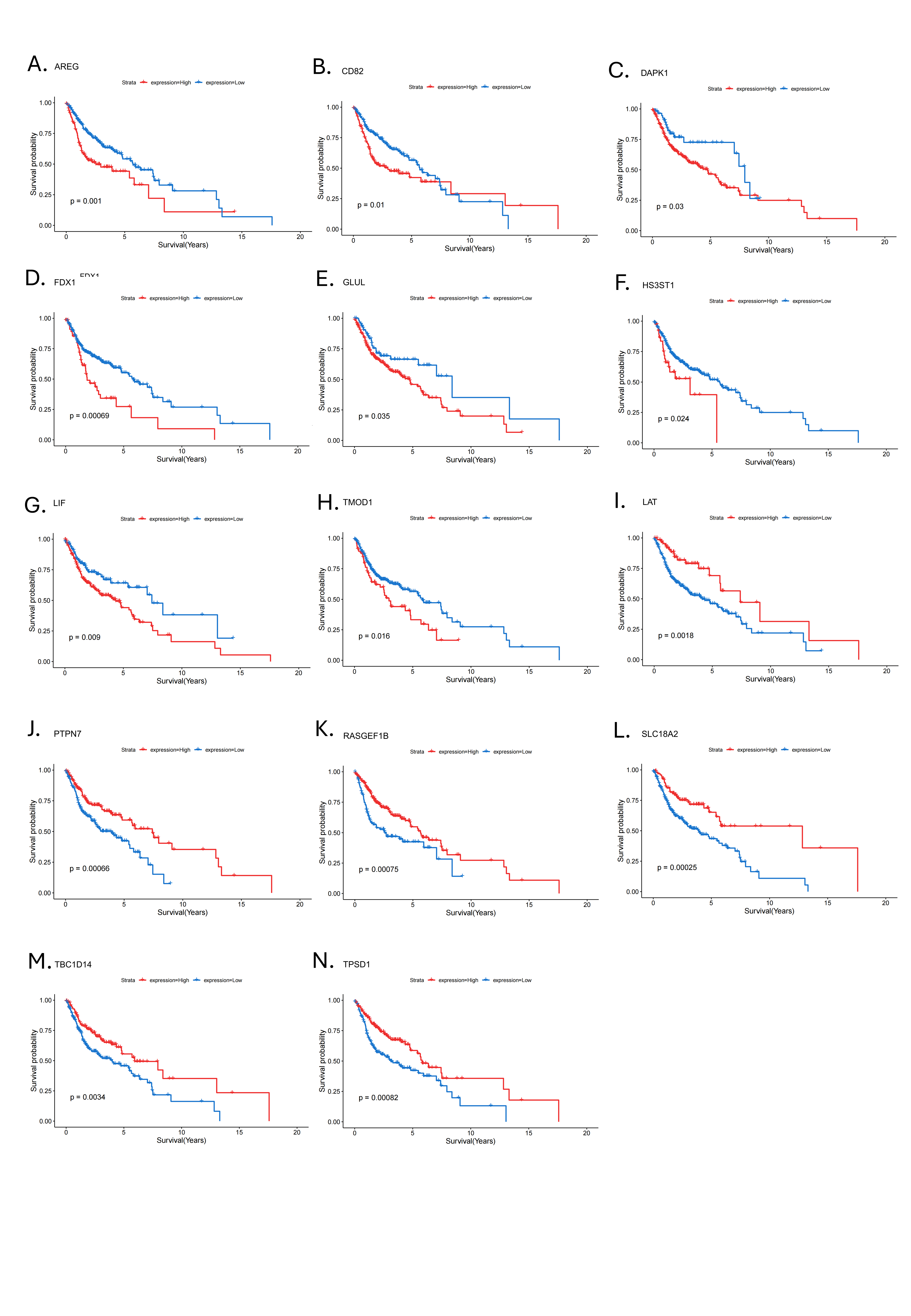


**Supplementary Figure 2.** **(A-N)** Kaplan-Meier curves of OS stratified by high expression versus low expression of 14 MRGs.
